# A Combination Scheme of Pure Strapdown and Dual-Axis Rotation Inertial Navigation Systems

**DOI:** 10.3390/s23063091

**Published:** 2023-03-14

**Authors:** Hongyang He, Feng Zha, Feng Li, Qiushuo Wei

**Affiliations:** 1 Department of Navigation, Naval University of Engineering, Wuhan 430033, China; 2 Naval Research Institute of PLA, Beijing 100161, China

**Keywords:** dual inertial navigation system, strapdown inertial navigation system, rotation inertial navigation system, Kalman filter, attitude error

## Abstract

Compared with the strapdown inertial navigation system (SINS), the rotation strapdown inertial navigation system (RSINS) can effectively improve the accuracy of navigation information, but rotational modulation also leads to an increase in the oscillation frequency of attitude errors. In this paper, a dual-inertial navigation scheme that combines the strapdown inertial navigation system and the dual-axis rotation inertial navigation system is proposed, which can effectively improve the attitude error accuracy in the horizontal direction by using the high-position information of the rotation inertial navigation system and the stability characteristics of the attitude error of the strapdown inertial navigation system. Firstly, the error characteristics of the strapdown inertial navigation system and the rotation strapdown inertial navigation system are analyzed, and then the combination scheme and Kalman filter are designed according to the error characteristics, and finally, the simulation experiment shows that the pitch angle error of the dual inertial navigation system is reduced by more than 35% and the roll angle error is reduced by more than 45% compared with the rotation strapdown inertial navigation system. Therefore, the combination scheme of double inertial navigation proposed in this paper can further reduce the attitude error of the rotation strapdown inertial navigation system, and at the same time, the two sets of inertial navigation systems can also enhance the reliability of ship navigation.

## 1. Introduction

The strapdown inertial navigation system can provide stable and reliable navigation information for passenger/cargo ships, submarines, ships and other military carriers [1,2,3,4]. The disadvantage is that the long-endurance navigation accuracy is limited by the accuracy of its own inertial device, and the improvement of the accuracy of the inertial device is limited by research costs, manufacturing materials and the manufacturing process [5,6,7,8]. In addition, online calibration technology and rotation modulation technology can reduce navigation errors at the system level. Among them, the rotation modulation technology not only ensures the concealment of the inertial navigation system, but also can reduce the error of the inertial navigation system, so the rotation strapdown inertial navigation system has been widely studied and applied [9,10,11,12,13]. American company Sperry proposed a dual-axis rotation scheme with ±180° rotation of the azib axis and the roll axis, which can modulate the main error of the inertial device, and the scheme has been applied to MK49WSN-7A and other rotation inertial navigation systems [14]; Baolun Yuan et al. designed a 16-position dual-axis rotation modulation scheme, which effectively modulates the constant drift of inertial devices and improves the positioning accuracy of the inertial navigation system [15]; Zhinong Ji et al. further improved the rotation scheme on the basis of the former scheme, and on the basis of completely modulating the constant drift, the scale factor error can be modulated, which improves the accuracy of navigation information [16]; Huiying Fan et al. designed an integrated scheme of 48-position rotation modulation and online calibration, which greatly improved the accuracy of the inertial navigation system [17].

In order to ensure the reliability of the inertial navigation system, most of the early large and medium-sized ships used two or more sets of inertial navigation systems, and the subsystem was the backup system of the main system, so the system accuracy could not be improved. With the development of inertial navigation systems, the research of dual inertial navigation combination began to focus on and improve the navigation accuracy of the system [18]. Liu Weiren et al. used two sets of dual-axis rotation strapdown inertial navigation systems to design a dual inertial navigation information fusion scheme with different rotation strategies, which reduces the velocity error by estimating the error of the main inertial navigation device, and then improves the alignment accuracy of the sub-inertial conduction [19]. Wang Lin et al. fused the navigation information of the single-axis rotation strapdown inertial navigation system and the dual-axis rotation strapdown inertial navigation system to effectively improve the positioning accuracy of the dual-axis inertial navigation system [20]; Wu Qi et al. used the redundant information of two sets of dual rotation strapdown inertial navigation systems to improve the observability of inertial device bias [21]; Cui Jiarui et al. proposed an online estimation and self-correction scheme for the scale factor error of optical fiber gyro on the basis of two sets of three-axis rotation strapdown inertial navigation systems, which effectively improved the positioning accuracy of the optical fiber gyro navigation inertial navigation system [22].

Compared with the strapdown inertial navigation system, the rotation strapdown inertial navigation system can modulate the constant value error of the inertial device in the horizontal direction, so the positioning error of the rotation strapdown inertial navigation system is greatly reduced. The rotational modulation effect is manifested as an average attitude error of zero in the rotational modulation cycle, and the attitude oscillation frequency of the rotation strapdown inertial navigation system is significantly increased compared with that of the strapdown inertial navigation system. In this paper, the combination of two sets of rotation strapdown inertial navigation systems is carried out by using the high positioning accuracy of the rotation strapdown inertial navigation system and the stable output characteristics of the strapdown inertial navigation system. Firstly, the error characteristics of the two inertial navigation systems are analyzed, and then the dual inertial navigation scheme is designed, and the combined navigation correction of the strapdown inertial navigation system is carried out by using the more accurate position information obtained by the rotation strapdown inertial navigation system as a reference, and finally, the experimental verification shows that the dual inertial navigation combination scheme can improve the horizontal attitude accuracy of the rotation strapdown inertial navigation system. Compared with the rotation inertial navigation system, the pitch angle error of the dual inertial navigation system is reduced by more than 35% and the roll angle error is reduced by more than 45%, and the equipment arrangement of the two inertial navigation systems greatly improves the reliability of the navigation system.

## 2. Error Characterization

### 2.1. Error Characteristics of SINS

The error of the strapdown inertial navigation system mainly includes the attitude error, velocity error and position error, and the error is mainly caused by the error of the inertial device, and the error of the inertial device mainly includes constant value error, scale factor error and installation error. Without considering ambient temperature and other component errors, the attitude error, velocity error, and position error equations for a strapdown inertial navigation system are shown below.

(1) $$\overset{\cdot }{\phi }=-\omega_{in}^{n}\times \phi +\delta \omega_{in}^{n}-C_{b}^{n}\delta \omega_{ib}^{b}$$

(2) $$\begin{array}{cc} \delta \overset{\cdot }{v}= & f^{n}\times \phi +C_{b}^{n}\delta f^{b}-(2\omega_{ie}^{n}+\omega_{en}^{n})\times \delta v\\ & -(2\delta \omega_{ie}^{n}+\delta \omega_{en}^{n})\times v-\delta g^{n} \end{array}$$

(3) $$\{\begin{array}{c} \delta \dot{L}=\frac{\delta v_{N}}{R_{M}}\\ \delta \dot{\lambda }=\frac{\delta v_{E}}{R_{N}\cos L}+\delta L\frac{v_{E}}{R_{N}}\tan L\sec L \end{array}$$

Among them, i represents the geocentric inertial frame, n represents the navigation frame, b represents the body frame, and e represents the earth frame. ϕ represents the attitude angle error of the mathematical platform, v represents the velocity, L represents the latitude, λ represents the longitude, $\omega_{in}^{n}$ represents the angular rate of rotation of the navigation frame relative to the inertial frame, $\delta \omega_{ib}^{b}$ represents the angular velocity error measured by the gyroscope, $\delta f^{b}$ represents the acceleration error measured by the accelerometer, $C_{b}^{n}$ represents the transformation matrix from the body frame to the navigation frame, $C_{b}^{n}=I$ is usually defined in the strapdown inertial navigation system, I is the unit matrix, $\omega_{ie}^{n}$ is the angular velocity of the earth’s rotation in the navigation frame, $\omega_{en}^{n}$ is the projection of the angular velocity of rotation of the navigation frame relative to the earth frame in the navigation frame, g is the local gravitational acceleration, $R_{M}$ is the radius of curvature of the meridian ring, and $R_{N}$ is the radius of curvature of the unitary circle.

**Figure 1:**
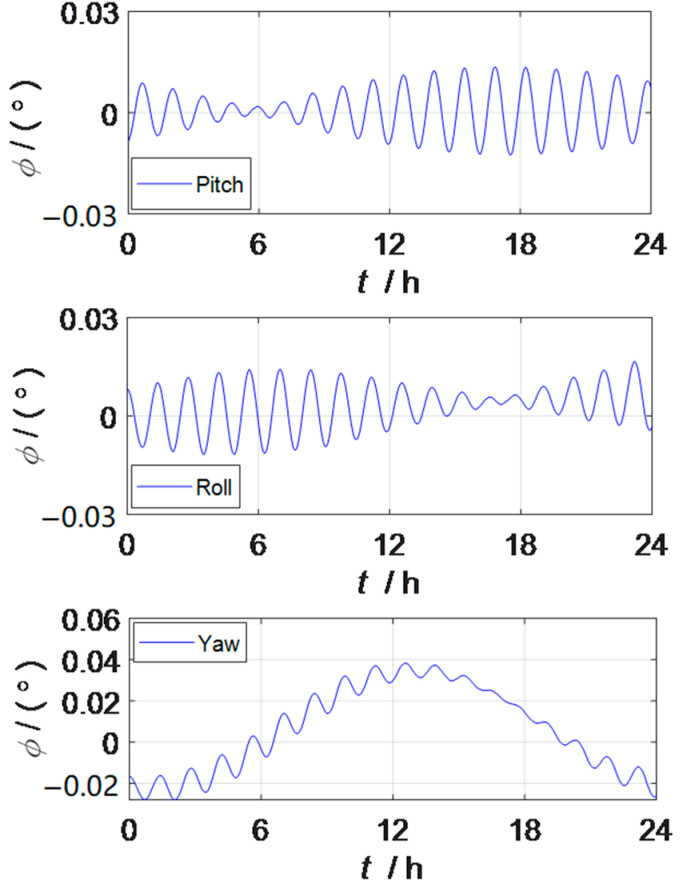
Attitude error of the strapdown inertial navigation system.

**Figure 2:**
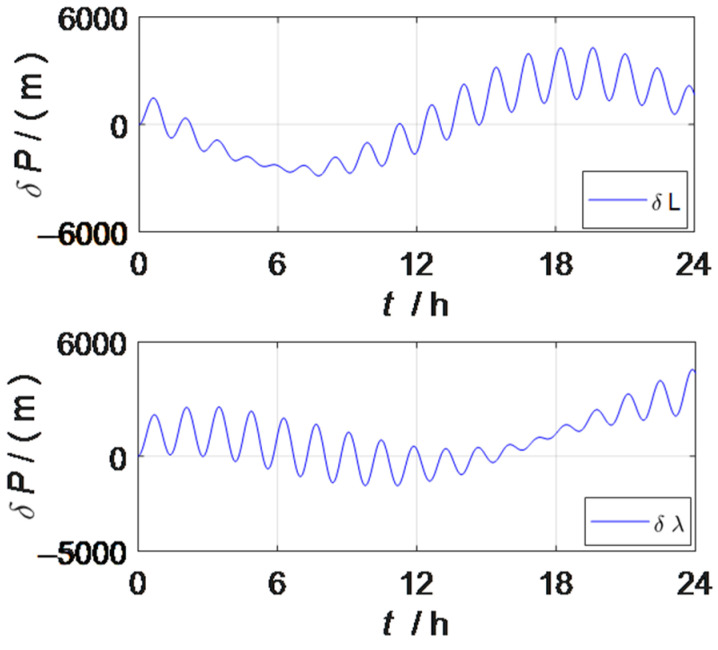
Position error of the strapdown inertial navigation system.

When the strapdown inertial navigation system is excited by the constant value error and random error of the inertial device, the attitude error and position error curves of the strapdown inertial navigation system can be observed as shown in Figure 1 and Figure 2. The oscillation period of the two error curves is mainly manifested as the Shura oscillation period.

### 2.2. Error Characteristics of Dual-Axis RSINS

In contrast to the structure of the strapdown inertial navigation system, the composition of the rotation inertial navigation system also includes a rotation mechanism, and the inertial device error can be periodically modulated by designing a reasonable rotational modulation scheme. Due to the existence of rotation, it is necessary to define a rotational frame to express rotational motion, where the rotational frame can be understood as an IMU frame. The attitude error and velocity error of the rotation inertial navigation system are shown below.

(4) $$\overset{\cdot }{\phi }=-\omega_{in}^{n}\times \phi +\delta \omega_{in}^{n}-C_{b}^{n}C_{p}^{b}\delta \omega_{ip}^{p}$$

(5) $$\begin{array}{cc} \delta \overset{\cdot }{v}= & f^{n}\times \phi +C_{b}^{n}C_{p}^{b}\delta f^{p}-(2\omega_{ie}^{n}+\omega_{en}^{n})\times \delta v\\ & -(2\delta \omega_{ie}^{n}+\delta \omega_{en}^{n})\times v-\delta g^{n} \end{array}$$

where p represents the rotation frame, $C_{p}^{b}$ represents the transformation matrix between the rotation frame and the body frame, and periodically transforms with the rotation mechanism of the rotation mechanism, where $C_{p}^{b}=I$ at the initial moment.

The angular velocity error $\delta \omega_{ib}^{n}$ measured by the gyroscope and the acceleration error $\delta f^{n}$ measured by the accelerometer are caused by the inertial device constant value error, scale factor error, and installation error, and the specific expressions are as follows.

(6) $$\delta \omega_{ib}^{n}=C_{b}^{n}C_{p}^{b}\delta \omega_{ip}^{p}=C_{b}^{n}C_{p}^{b}\left[\left(\delta K_{g}+\delta A_{g}\right)\omega_{ip}^{p}+\varepsilon^{p}\right]$$

(7) $$\delta f^{n}=C_{b}^{n}C_{p}^{b}\delta f^{p}=C_{b}^{n}C_{p}^{b}\left[\left(\delta K_{a}+\delta A_{a}\right)f^{p}+\nabla^{p}\right]$$

Among them, ε and ∇ are the constant value errors of the gyroscope and accelerometer, respectively; $\delta K_{g}$ and $\delta K_{a}$ are the scale factor errors for gyroscopes and accelerometers, respectively; $\delta A_{g}$ and $\delta A_{a}$ are the installation errors for gyroscopes and accelerometers, respectively; $\omega_{ip}^{p}$ and $f^{p}$ are the ideal outputs for gyroscopes and accelerometers in rotational frames, respectively.

Taking the gyroscope output error as an example, the influence of rotational modulation on the error characteristics of the rotational inertial navigation system is analyzed, and it is assumed that under static base navigation, the body frame coincides with the navigation frame, that is, $C_{b}^{n}=I$.

1. Constant value error modulation principle

Assuming that the output error of the gyroscope under the rotation inertial navigation system is only caused by the constant value error, when the IMU rotates around the *z*-axis at speed ω, the systematic error caused by the constant value error is as follows:

(8) $$\delta \omega_{ib}^{n}=C_{b}^{n}C_{p}^{b}\varepsilon^{p}=\left[\begin{array}{c} \varepsilon_{x}^{p}\cos\omega t-\varepsilon_{y}^{p}\sin\omega t\\ \varepsilon_{x}^{p}\sin\omega t+\varepsilon_{y}^{p}\cos\omega t\\ \varepsilon_{z}^{p} \end{array}\right]$$

As can be observed from Equation (8), the components of the gyroscope constant value error in the x- and y-axes are modulated to the form of sine and cosine, and the $\delta \omega_{ib}^{n}$ integral gives the attitude error, and since ε is a constant value, the integral of the sine and cosine functions multiplied by the constant value in a period is zero, as shown in Equation (9).

(9) ϕ=∫02πωδωibndt=∫02πω[εxpcosωt−εypsinωtεxpsinωt+εypcosωtεzp]dt=[00εzp2πω]

Similarly, when the IMU rotates around the *x*-axis or *y*-axis, rotation modulation can modulate the constant value error perpendicular to the axis direction to the sine and cosine form, without causing attitude errors, and the constant value error in the direction of the rotation axis cannot be modulated. In a dual-axis rotation inertial navigation system, the IMU rotates alternately around the *x*-axis or *z*-axis, and the constant value error of the three axes can be intermittently modulated into a periodic form, so that the constant value error does not cause attitude errors.

2. Scale factor error and installation error modulation principle

The influence of the coupling of rotational motion with scale factor errors and installation errors on the system can be expressed as follows:

(10) $$\delta \omega_{ib}^{n}=C_{b}^{n}C_{p}^{b}\left(\delta K_{g}+\delta A_{g}\right)\omega_{bp}^{p}$$

The different rotation schemes $C_{p}^{b}$ and $\omega_{bp}^{p}$ are different, and the coupling errors are also different, so a reasonable rotation modulation scheme can not only modulate the constant value error, but also reduce the influence of the scale factor error, installation error and IMU rotational motion coupling error on the inertial navigation system as much as possible.

**Figure 3:**
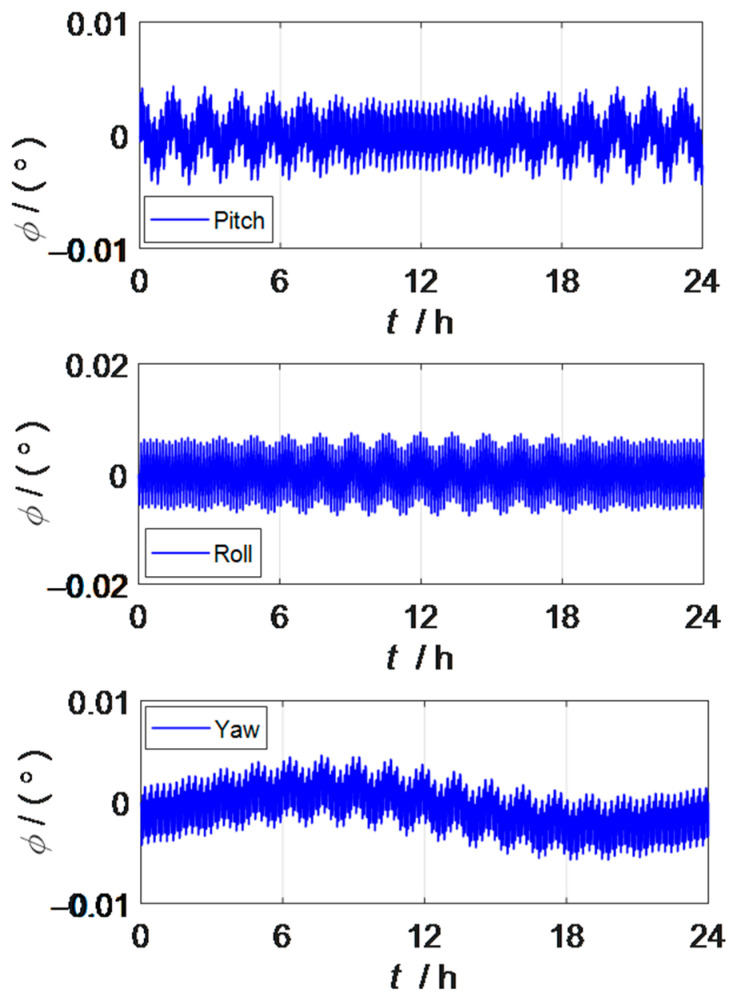
Attitude error of the rotation strapdown inertial navigation system.

**Figure 4:**
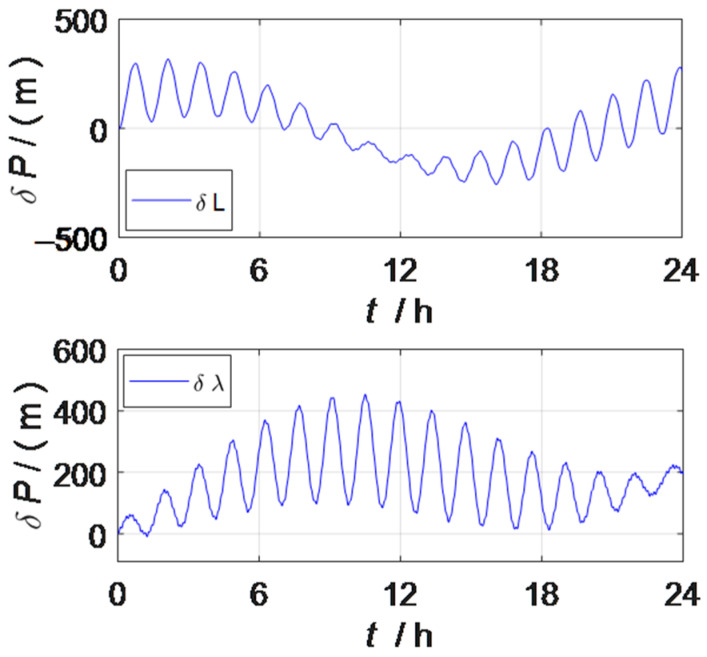
Position error of the rotation strapdown inertial navigation system.

The above section introduces the modulation principle of inertial device errors in rotation inertial navigation systems, and explains the error transfer characteristics in rotation inertial navigation systems. It can be observed that unlike the strapdown inertial navigation system, the rotation inertial navigation system is not only affected by the constant value error of the inertial device, but also greatly affected by the coupling error caused by the rotational motion, the scale factor error and installation error. When the rotation inertial navigation system is excited by the constant value error, random error, scale factor error and installation error of the inertial device, the attitude error and position error of the rotation inertial navigation system can be observed as shown in Figure 3 and Figure 4. Figure 3 shows the characteristic of the attitude error amplitude curve oscillation frequency increasing under the action of rotational modulation.

### 2.3. Comparison of Error Characteristics of Two INSs

Figure 1 and Figure 3 are the attitude error curves of the strapdown inertial navigation system and the rotation inertial navigation system, respectively, and the pitch angle accuracy of the rotation inertial navigation system is improved by more than 64%, the roll angle accuracy is increased by more than 54%, and the heading angle accuracy is increased by more than 88%, so the attitude information accuracy in the three directions has been greatly improved by rotation modulation. From the perspective of the vibration frequency of the attitude error curve, the oscillation frequency of the attitude error curve of the rotation inertial navigation system is higher than that of the strapdown inertial navigation system, because the dual-axis rotation inertial navigation system adopts a 16-position rotary stop rotation scheme, which can modulate the constant value error of the inertial device to the form of a zero mean period in one rotation cycle to achieve the effect of automatic compensation. The constant value error, scale factor error, and mounting error of gyroscopes and accelerometers that are self-compensated by rotational modulation in the inertial navigation system do not disappear, but the impact on the system is averaged. At the same time, it shows that the navigation information output of the strapdown inertial navigation system is relatively stable.

Figure 2 and Figure 4 are the position error curves of the strapdown inertial navigation system and the rotation inertial navigation system, respectively, and the latitude error of the rotation inertial navigation system is reduced by more than 90% and the longitude error is reduced by more than 89%, compared with the strapdown inertial navigation system. The rotation inertial navigation system shows excellent positioning ability.

In summary, the strapdown inertial navigation system has the characteristics of stable navigation information output compared with the rotation inertial navigation system, and the rotation inertial navigation system has the characteristics of higher navigation accuracy than the strapdown inertial navigation system. Therefore, the combination of the strapdown inertial navigation system and rotation inertial navigation system can be studied, so that the advantages of the two inertial navigation systems complement each other.

## 3. Combined Method of Dual Inertial Navigation System

### 3.1. Dual Inertial Navigation Combination Scheme

Combined navigation is usually a combination of navigation systems with different characteristics, using the advantages of both parties to overcome the shortcomings of both sides, so combined navigation technology is an effective means to improve the overall performance of the navigation system, and common combined navigation methods include SINS/GPS (global positioning systems, GPS) combined navigation, SINS/DVL (Doppler log) combined navigation, etc. [23,24,25,26]. Among them, the SINS/GPS combined navigation method loses the concealment characteristics of the inertial navigation system, and the Doppler meter in the SINS/DVL combined navigation method does not need to radiate energy outward, so the combined method ensures the concealment characteristics of the inertial navigation system. The dual inertial guidance combined navigation method uses the combination of two sets of inertial navigation systems to also ensure the concealment characteristics. With the above comparison and analysis of the error characteristics of the strapdown inertial navigation system and the rotation strapdown inertial navigation system, the rotation strapdown inertial navigation system shows the advantages of a small error amplitude, and the advantage of the strapdown inertial navigation system is reflected in the fact that the attitude error is more stable than that of the rotation strapdown inertial navigation system. Combined navigation using the advantages of the two inertial navigation systems can further improve the accuracy of navigation information. At the same time, the combination of dual inertial navigation enhances the reliability of navigation compared to a single inertial navigation system.

**Figure 5:**
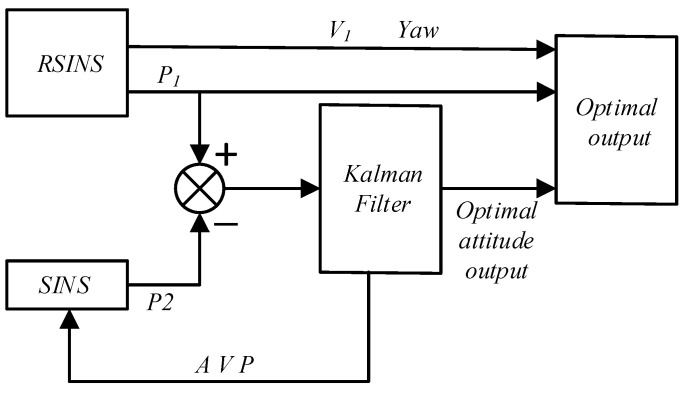
Dual inertial navigation combination scheme.

As shown in Figure 5, the dual inertial navigation scheme consists of a rotation inertial navigation system and a strapdown inertial navigation system, and the two inertial navigation systems have the same initial conditions and work at the same time, so the two inertial navigation systems can become each other’s navigation backup systems, assuming that the position information output by the rotation strapdown inertial navigation system is true, the position information of the two sets of inertial navigation output is used as the observation measurement of the Kalman filter, and the estimated attitude, velocity and position information of the Kalman filter output is fed back to the strapdown inertial navigation system, while the horizontal attitude is taken as the optimal attitude of the dual inertial navigation system. The rest of the optimal navigation information is taken from the navigation information output by the rotation inertial navigation system.

### 3.2. Kalman Filter Design

The Kalman filter is one of the most commonly used mathematical methods in combined navigation, which can fuse the navigation attitude, velocity and position information calculated by the inertial navigation system with the attitude, speed and position information output by other auxiliary navigation systems, filter out the deviations of the system, and provide the optimal value of navigation system information [27]. The combined navigation system state equation can be expressed as follows:

(11) $$\dot{X}\left(t\right)=F\left(t\right)X\left(t\right)+G\left(t\right)W\left(t\right)$$

where X(t) is a 15-dimensional state vector, as shown in Equation (12), F(t) is the state transition matrix, which can be expressed by Equations (1)–(3), and G(t) is the noise distribution matrix of the strapdown inertial navigation system; W(t) is the noise matrix of the strapdown inertial navigation system.

(12) $$\begin{array}{cc} X\left(t\right)= & [\begin{array}{cccc} \varphi_{E} & \varphi_{N} & \varphi_{U} & \begin{array}{ccc} \begin{array}{cc} \delta v_{E} & \delta v_{N} \end{array} & \delta v_{U} & \delta L \end{array} \end{array}\\ & {\begin{array}{cccc} & \delta \lambda & \delta h & \begin{array}{cccc} \varepsilon_{x} & \varepsilon_{y} & \varepsilon_{z} & \begin{array}{ccc} \nabla_{x} & \nabla_{y} & \nabla_{z} \end{array} \end{array} \end{array}]}^{T} \end{array}$$

The combined navigation system observation equation is as follows:

(13) Z(t)=H(t)X(t)+V(t)

where Z(t) is the observation vector, H(t) is the systematic observation matrix, and V(t) is the observation noise matrix.

The difference between the positions of the two sets of inertial guided outputs is the observational measurement, so the observation vector Z(t) and the systematic observation matrix H(t) can be written as follows:

(14) $$Z\left(t\right)=\left[\begin{array}{c} L_{RSINS}-L_{SINS}\\ \lambda_{RSINS}-\lambda_{SINS}\\ h_{RSINS}-h_{SINS} \end{array}\right]$$

(15) $$H\left(t\right)=\left[\begin{array}{ccc} 0_{3\times 6} & I_{3\times 3} & 0_{3\times 6} \end{array}\right]$$

Most of the stochastic system models established in practical application are time-continuous, and continuous-time models need to be discretized when using computers for navigation calculations and Kalman filter estimation. Discretization of the established Kalman filter model can be expressed as follows:

(16) $$\{\begin{array}{l} X_{k}=F_{k/k-1}X_{k-1}+\Gamma_{k/k-1}W_{k-1}\\ Z_{k}=H_{k}X_{k}+V_{k} \end{array}$$

The Kalman filter algorithm is an iterative algorithm, and the setting of the initial parameters is a key factor in the filter model. The Kalman filter uses the observed attitude, velocity, and position information at the current moment with the estimated value obtained after filtering at the previous moment, and provides the optimal estimate of variables such as the navigation attitude, velocity, and position through layer by layer recursion. The prediction and recurrence process of the Kalman filter is as follows:

State one-step prediction:

(17) $${\hat{X}}_{k/k-1}=F_{k/k-1}{\hat{X}}_{k-1}$$

State one-step prediction mean square error matrix:

(18) $$P_{k/k-1}=F_{k/k-1}P_{k-1}F_{k/k-1}^{T}+\Gamma_{k-1}Q_{k-1}\Gamma_{k-1}^{T}$$

Filter gain calculation:

(19) $$K_{k}=P_{k/k-1}H_{k}^{T}{\left(H_{k}P_{k/k-1}H_{k}^{T}+R_{k}\right)}^{-1}$$

State estimation:

(20) $${\hat{X}}_{k}={\hat{X}}_{k/k-1}+K_{k}\left(Z_{k}-H_{k}{\hat{X}}_{k/k-1}\right)$$

Mean square error of state estimation:

(21) $$P_{k}=\left(I-K_{k}H_{k}\right)P_{k/k-1}$$

where $Q_{k}$ represents the mean square error matrix of the output white noise of the gyroscope and accelerometer, and $R_{k}$ represents the variance matrix of the observation noise. In general, it is required that $Q_{k}$ is non-negative definite and $R_{k}$ is positive definite.

## 4. Combined Simulation Verification of Dual INS

### 4.1. Experimental Conditions

Design simulation experiments verify the navigation effect of the dual inertial navigation combination scheme of pure strapdown inertial navigation systems and dual-axis rotation inertial navigation systems. The setting of the simulation experimental conditions is consistent with the current experience level of high-precision inertial navigation systems, and unlike the strapdown inertial navigation system, the scale factor error, installation error and rotational motion in the rotation inertial navigation system will produce coupling errors, so in addition to the constant value error in the simulation of the rotation inertial navigation system, it is necessary to increase the scale factor error and installation error. The specific simulation experiment parameter settings are as follows:

(1) Strapdown inertial navigation system error source: gyroscope constant value error 0.001${}^{\circ }/h$, random error 0.0003${}^{\circ }/\sqrt{h}$, accelerometer constant value error 10 μg and random error 1 $\mu g/\sqrt{Hz}$;
(2) Rotation strapdown inertial navigation system error sources: gyroscope constant value error 0.001${}^{\circ }/h$, random error 0.0003${}^{\circ }/\sqrt{h}$, accelerometer constant value error 10 μg, random error 1 $\mu g/\sqrt{Hz}$, gyroscope scale factor error 5 ppm, gyroscope installation error 5″, accelerometer scale factor error 10 ppm and accelerometer installation error 10″;
(3) Initial latitude and longitude: 114°, 30°;
(4) Sampling time: 0.1 s.

The dual-axis rotation inertial navigation system adopts a 16-position rotation scheme, and the initial X-axis, Y-axis and Z-axis point east, north and upwards, respectively, and the specific rotation scheme is as follows:

(1) Rotate 180° along the positive Z-axis and stay for 30 s.
(2) Rotate 180° along the negative X-axis and stay for 30 s.
(3) Rotate 180° along the positive X-axis and stay for 30 s.
(4) Rotate 180° along the negative Z-axis and stay for 30 s.
(5) Rotate 180° along the negative X-axis and stay for 30 s.
(6) Rotate 180° along the positive Z-axis and stay for 30 s.
(7) Rotate 180° along the negative Z-axis and stay for 30 s.
(8) Rotate 180° along the positive X-axis and stay for 30 s.
(9) Rotate 180° along the negative Z-axis and stay for 30 s.
(10) Rotate 180° along the positive X-axis and stay for 30 s.
(11) Rotate 180° along the negative X-axis and stay for 30 s.
(12) Rotate 180° along the positive Z-axis and stay for 30 s.
(13) Rotate 180° along the positive X-axis and stay for 30 s.
(14) Rotate 180° along the negative Z-axis and stay for 30 s.
(15) Rotate 180° along the positive Z-axis and stay for 30 s.
(16) Rotate 180° along the negative X-axis and stay for 30 s.

The angular velocity of rotation for the rotation scheme is set to 6°/s [28].

### 4.2. Experimental Results

**Figure 6:**
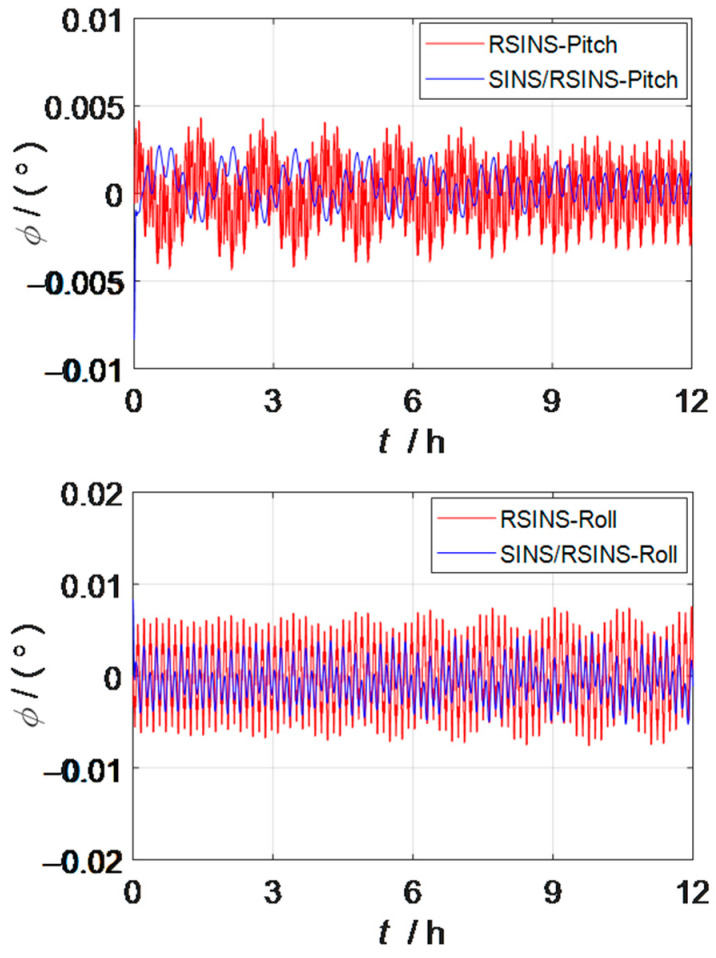
Comparison curve of dual INS combination scheme and RSINS horizontal attitude error.

The comparison curve of the horizontal attitude error between the dual inertial navigation combination system and the rotation inertial navigation system within 24 h is shown in Figure 6. The maximum pitch angle error of the dual inertial navigation system is reduced by 0.00154° compared with the rotation inertial navigation system, and the error reduction accounts for more than 35% of the pitch angle error of the rotation inertial navigation system. The maximum roll angle error of the dual inertial navigation system is 0.003542° lower than that of the rotation inertial navigation system, and the error reduction accounts for more than 45% of the roll angle error of the rotation inertial navigation system. Therefore, the combined scheme of dual inertial navigation proposed in this paper can improve the accuracy of the horizontal attitude angle. In the simulation experiment, it is also found that the shortcomings of the dual inertial guidance combination scheme proposed in this paper are not as accurate as the heading angle of the rotation inertial navigation system, so it is not shown in this paper. This issue will be considered in follow-up studies.

## 5. Conclusions

The rotation inertial navigation system effectively improves the navigation accuracy of the inertial navigation system due to the rotation modulation technology, but it increases the fluctuation frequency of the system attitude error curve, which has a negative impact on the attitude accuracy of the system. In this paper, a dual inertial navigation scheme of a strapdown inertial navigation system and dual-axis rotation strapdown inertial navigation system is proposed, which can effectively improve the horizontal attitude accuracy by using the stability characteristics of the attitude information of the strapdown inertial navigation system and the high-precision position information output by the rotation strapdown inertial navigation system. Simulation experiments show that the pitch angle error of the dual inertial navigation system is reduced by more than 35% and the roll angle error is reduced by more than 45%, compared with the rotation strapdown inertial navigation system. This verifies that the combined scheme of dual inertial navigation proposed in this paper is an effective method to further reduce the attitude error of the rotation strapdown inertial navigation system, and at the same time, the two sets of inertial navigation systems also enhance the reliability of ship navigation.

## Data Availability

This study did not report any data.
